# MitoQ Ameliorates Diabetic Cardiomyopathy by Inhibiting the mtROS–TXNIP–NLRP3 Pathway

**DOI:** 10.1155/mi/1505350

**Published:** 2026-07-21

**Authors:** Shaohuan Qian, Junjie Leng, Zhuoya Yao, Chao Shi, Wei Zhang

**Affiliations:** ^1^ Department of Cardiovascular Medicine, The First Affiliated Hospital of Bengbu Medical University, Bengbu City 233000, Anhui, China, bbmc.edu.cn; ^2^ Department of Cardiac Surgery, The First Affiliated Hospital of Bengbu Medical University, Bengbu City 233000, Anhui, China, bbmc.edu.cn

**Keywords:** diabetic cardiomyopathy, MitoQ, NOD-like receptor protein 3, thioredoxin-interacting protein

## Abstract

**Purpose:**

To determine whether mitoquinone mesylate (MitoQ) could treat diabetic cardiomyopathy (DCM) by inhibiting the mitochondrial reactive oxygen species (mtROS)/thioredoxin (TRX)‐interacting protein (TXNIP)/NOD‐like receptor protein 3 (NLRP3) pathway.

**Methods:**

In vivo DCM models were established using a high‐fat diet combined with streptozotocin injection in mice, whereas in vitro models were generated by exposing AC16 cardiomyocytes to high glucose. Immunohistochemistry (IHC) and western blotting were used to analyze the expression levels of TXNIP, NLRP3, Caspase‐1, and other related proteins in cardiac tissue and cardiomyocytes stimulated with high glucose. mtROS fluorescence staining was used to analyze whether MitoQ could alleviate the generation of ROS in mitochondria in a high‐glucose environment. Co‐IP experiments were used to analyze whether high glucose stimulation promoted the interaction between TXNIP and NLRP3 and induced NLRP3 inflammasome activation.

**Results:**

Diabetic mice exhibited increased oxidative stress, enhanced mtROS accumulation, activation of the TXNIP/NLRP3 inflammasome pathway, myocardial fibrosis, and impaired cardiac function. High‐glucose stimulation in AC16 cells promoted dissociation of TXNIP from TRX, enhanced TXNIP–NLRP3 interaction, and increased expression of downstream pyroptosis‐related proteins, including NT‐gasdermin D (GSDMD), Caspase‐1, and cleaved interleukin‐1β (IL‐1β). MitoQ treatment reduced mtROS production, restored mitochondrial membrane potential (MMP), inhibited TXNIP–NLRP3 interaction, and suppressed inflammasome activation both in vivo and in vitro. Moreover, TXNIP knockdown further enhanced the protective effects of MitoQ, confirming the critical role of the mtROS/TXNIP/NLRP3 axis.

**Conclusion:**

MitoQ attenuates diabetic myocardial injury by inhibiting mtROS accumulation and suppressing TXNIP/NLRP3 inflammasome activation. Targeting the mtROS/TXNIP/NLRP3 signaling pathway may represent a promising therapeutic strategy for DCM.

## 1. Introduction

Diabetic cardiomyopathy (DCM) is a chronic and progressive heart muscle disease that slowly impairs heart function and ultimately results in serious complications such as heart failure [1, 2]. With the global rise in diabetes prevalence, the incidence of DCM continues to increase, highlighting the urgent need for effective preventive and therapeutic strategies. Although the mechanisms underlying DCM are not fully elucidated, insulin resistance, lipid peroxidation, and oxidative stress are recognized as major contributors [3, 4]. These factors promote inflammatory forms of programmed cell death, including pyroptosis, thereby contributing to cardiac fibrosis and progressive cardiac dysfunction.

Pyroptosis is a form of inflammatory programmed cell death mediated by gasdermin (GSDM), characterized by inflammasome activation and the release of pro‐inflammatory cytokines [5]. The NOD‐like receptor protein 3 (NLRP3) inflammasome plays a central role in pyroptosis. Upon activation, it promotes Caspase‐1 cleavage and activation [6]. Activated Caspase‐1 subsequently cleaves GSDMD, releasing the N‐terminal fragment that forms pores in the plasma membrane, resulting in membrane rupture and the release of inflammatory cytokines, including interleukin‐1β (IL‐1β) and IL‐18, thereby initiating pyroptosis [7, 8]. Thioredoxin (TRX)‐interacting protein (TXNIP) is a critical signaling molecule linking oxidative stress to inflammasome activation [9, 10]. Under physiological conditions, TRX binds to TXNIP and suppresses its activity. However, intracellular reactive oxygen species (ROS) promote the dissociation of TXNIP from TRX, facilitating its binding to NLRP3 and triggering inflammasome assembly [11, 12]. In DCM, insulin deficiency or resistance shifts myocardial energy metabolism toward increased fatty acid oxidation [13]. Chronic reliance on fatty acids disrupts the mitochondrial oxidative balance and enhances acylcarnitine accumulation, leading to excessive mitochondrial ROS (mtROS) generation [14]. More than two‐thirds of ROS in cardiomyocytes originate from mitochondria [15]. mtROS facilitates NLRP3 inflammasome assembly, thereby exacerbating pyroptosis in cardiomyocytes [16]. Therefore, excessive mtROS generation is a pivotal driver of NLRP3 activation, and reducing mtROS production may represent a key therapeutic strategy for inhibiting pyroptosis.

Mitoquinone mesylate (MitoQ), a mitochondria‐targeted quinone derived from ubiquinone, functions as a selective mitochondrial antioxidant. Within mitochondria, MitoQ prevents lipid peroxidation, reduces malondialdehyde (MDA) production, and inhibits ROS accumulation [17]. Previous studies have demonstrated that MitoQ mitigates oxidative damage in various pathological conditions. Moreover, scavenging ROS with MitoQ confers cardioprotective effects in animal models of heart failure and ischemia–reperfusion injury [18, 19]. However, limited evidence exists regarding whether MitoQ suppresses NLRP3 activation in DCM by modulating the mtROS/TXNIP pathway and protecting cardiomyocytes from oxidative stress. Therefore, this study aimed to investigate whether MitoQ attenuates DCM through the regulation of the mtROS/TXNIP/NLRP3 signaling axis.

## 2. Materials and Methods

### 2.1. Experimental Reagents

The following reagents were purchased from the respective suppliers. MitoQ (845959) was purchased from Solarbio Life Science. Cleaved‐IL‐1β rabbit mAb (83186) and TXNIP rabbit mAb (14715) were obtained from Cell Signaling Technology, Inc. NLRP3 rabbit polyclonal antibody (A1038) was purchased from Abcam Biotechnology Co., Ltd. (Shanghai, China). Anti‐cleaved GSDMD (N‐terminal) antibody (A22523), FITC‐conjugated goat anti‐rabbit IgG (AS011), and Cy3‐conjugated goat anti‐rabbit IgG (AS007) were obtained from Abclon Biotechnology Co., Ltd. (Wuhan, China). One Step TUNEL Apoptosis Assay Kit (C1089), Total Superoxide Dismutase Assay Kit with WST‐8 (S0101S), Mitochondrial Membrane Potential Assay Kit with JC‐1 (C2006), Total Antioxidant Capacity Assay Kit (S0116), Cell Counting Kit‐8 (C037), and Calcein/PI Assay Kit (C2015S) were purchased from Beyotime Biotechnology Co., Ltd. (Shanghai, China).

### 2.2. Establishment of Animal Model

2‐month‐old male C57BL/6 mice were used in this study. Following a 6‐week period of high‐fat diet feeding, diabetes was induced in the mice by an intraperitoneal injection of STZ solution (35 mg/kg/d). Body weight and blood glucose levels in mice were regularly monitored. Establishing a diabetes mouse model was considered successful when the fasting blood glucose level of the mice exceeded 16.7 mmol/L. The MitoQ working solution was prepared by dissolving MitoQ (10 mg) in 1 ml of DMSO. In the treatment group, mitochondrial quinone was intraperitoneally injected at 5 mg/kg/d, whereas the control group received an equivalent volume of DMSO solution. The mice were randomly divided into four groups (*n* = 8 per group): normal control group (Con), without any treatment; MitoQ group, receiving intraperitoneal injection of MitoQ alone; diabetes group (DM), consisting of successfully induced diabetic mice; and diabetes + MitoQ group (DM + MitoQ), consisting of diabetic mice treated with intraperitoneal MitoQ. All animals were housed separately according to the group and maintained for 6 months. All experimental procedures were approved by the Animal Ethics Committee of the First Affiliated Hospital of Bengbu Medical College (Approval Number: byyfy‐kyk‐201911).

### 2.3. Tissue Processing and Histological Analysis

At the end of the experiment, the mice were euthanized by asphyxia using carbon dioxide. Hearts were excised, rinsed in PBS containing heparin, fixed in 4% paraformaldehyde, embedded in paraffin, and sectioned at 6 μm thickness. For histological evaluation, sections were deparaffinized, rehydrated, and subjected to hematoxylin–eosin (HE) staining to assess the myocardial morphology and Masson’s trichrome staining to evaluate fibrosis. For immunohistochemistry (IHC), antigen retrieval was performed using citrate buffer, followed by incubation with primary antibodies against TRX, TXNIP, NLRP3, Caspase‐1, and cleaved IL‐1β overnight at 4°C. After incubation with HRP‐conjugated secondary antibodies, staining was visualized using DAB and counterstained with hematoxylin. Images were captured under a light microscope. Quantitative analysis was performed using ImageJ software by measuring the positive staining area or integrated optical density in five randomly selected fields per section. Fibrosis was expressed as the percentage of the collagen‐positive area relative to the total myocardial area. All analyses were conducted in a blinded manner.

### 2.4. Cardiac Ultrasound

After the mice were anesthetized, their limbs were fixed on the experimental console, and the rodents were pulled backward with silk threads to keep the respiratory tract open. Echocardiography was performed using a Vevo1100 small animal ultrasound imaging platform and an MS400 ultrasound probe. The left ventricular end‐systolic diameter (LVESD), left ventricular end‐ diastolic diameter (LVEDD), left ventricular ejection fraction (LVEF), and fractional shortening were obtained.

### 2.5. Cell Culture

AC16 cardiomyocytes were used as the experimental cells. This cell line was preserved in our research group for long‐term passaging and cryopreservation. In the Con group, cardiomyocytes were cultured in a medium containing 5.5 mM glucose, whereas in the HG group, additional glucose was added to reach a final glucose concentration of 30 mM. Cells (1 × 10^5^ cells/well) were seeded in six‐well plates, and 3 nM TXNIP siRNA was transfected into AC16 cells using the Lipofectamine 3000 reagent (Thermo Fisher Scientific, Carlsbad, CA, USA). The siRNA vector was designed and synthesized by GenePharma Biological Company (Shanghai, China).

### 2.6. Terminal Deoxynucleotidyl Transferase‐Mediated dUTP Nick‐End Labeling (TUNEL) Assay

Cardiomyocyte injury in paraffin‐embedded myocardial sections was detected using a TUNEL assay kit according to the manufacturer’s instructions. Briefly, paraffin sections were deparaffinized in xylene, rehydrated through graded ethanol, and rinsed in distilled water. Sections were treated with proteinase K (20 μg/mL) at 37°C for 30 min, followed by thorough washing with PBS. The TUNEL reaction mixture containing TdT enzyme and fluorescent labeling solution was freshly prepared and added to the sections (50 μL per section). Samples were incubated at 37°C for 60 min in the dark. After washing with PBS, sections were mounted with an antifade mounting medium and observed under a fluorescence microscope.

### 2.7. Measurement of SOD Concentration

One hundred microliters of SOD sample preparation solution were added to every 1 × 10^6^ cells for complete lysis. Myocardial tissue was lysed by adding 100 µL of SOD sample preparation solution per 10 mg of tissue. The samples were centrifuged at 12,000 × g at 4°C for 5 min, and the resulting supernatant was collected for testing. Then, 160 µL of WST‐8 working solution was added to every 20 µL of the sample to be tested and incubated at 37°C for 30 min. Absorbance was measured at 450 nm using a microplate reader.

### 2.8. Total Antioxidant Capacity Determination

A total of 1 × 10^5^ cells, or 20 mg of myocardial tissue, was collected. PBS (200 µL) was added, and ultrasonic waves were used to disrupt the cells or tissues. The sample was then centrifuged at ~12,000 × g for 5 min at 4°C, and the supernatant was collected for subsequent determination. Peroxidase (20 μL) working solution was added to each detection well of the 96‐well plate. PBS (10 µL) was added to the blank control well, and 10 µL of the sample to be tested was added to the sample detection well. Next, 170 µL of total antioxidant capacity detection working solution was added to each well and mixed gently. After incubating for 6 min, the absorbance was measured at 425 nm.

### 2.9. Measurement of mtROS

A 1 mM Mito‐SOX detection reagent solution was diluted with a serum‐free cell culture base to prepare a working solution with a final concentration of 200 nM. The culture medium was aspirated into a culture dish, rinsed once with PBS, and a stained working solution was added to cover the cell surface. The dishes were placed in an incubator and incubated for 20 min. The staining solution was replaced with a fresh culture solution, and the peroxide content in the mitochondria was observed under a fluorescence microscope.

### 2.10. Detection of Mitochondrial Membrane Potential (MMP)

A Mitochondrial Membrane Potential Assay Kit with JC‐1 (Beyotime Biological Company, Shanghai, China) was used to detect changes in the MMP, reflecting the cell activity level. The culture medium was aspirated from the culture dish and washed once with PBS, after which 1 mL of JC‐1 staining working solution was added and mixed well. The cells were incubated for 20 min. After incubation, the supernatant was aspirated, and the cells were washed twice with JC‐1 staining buffer (1×). The cells were observed under a fluorescence or laser confocal microscope.

### 2.11. Western Blot Analysis

Total protein was extracted from myocardial tissues or cultured cells using RIPA lysis buffer supplemented with protease inhibitors. After centrifugation at 12,000 × g for 10 min at 4°C, the supernatants were collected, and protein concentrations were determined using a BCA protein assay kit. Equal amounts of protein were separated by SDS‐PAGE and transferred onto PVDF membranes. Membranes were blocked with 5% nonfat milk for 1 h at room temperature and then incubated overnight at 4°C with primary antibodies. After washing, membranes were incubated with appropriate HRP‐conjugated secondary antibodies for 1 h at room temperature. Protein bands were detected using an enhanced chemiluminescence (ECL) system. Band intensities were quantified using ImageJ software and normalized to the corresponding internal control. All experiments were independently performed three times.

### 2.12. Co‐Immunoprecipitation

After washing the cells twice with PBS, cardiomyocytes were collected using a cell scraper. SDS cell lysis buffer (200 μL) was added to the collected cells, mixed thoroughly, and placed on ice for 15 min to lyse. The lysed cells were centrifuged at 12,000 × g for 10 min, and the supernatant was collected. Protein A + G magnetic bead suspension (20 μL) was combined with 200 μL of antibody working solution and incubated for 1 h. After completion, the cells were washed three times with PBS. Antibody‐bound protein A + G magnetic beads (10 μL) were combined with 100 μL of lysate supernatant and incubated overnight at 4°C with slow shaking. The beads were separated using a magnetic rack. 1x SDS‐PAGE loading buffer (100 μL) was mixed with 20 μL of separated beads and boiled at 100°C for 5 min. The mixture was again placed on a magnetic stand, and the supernatant was collected for analysis.

### 2.13. Statistical Analysis

All quantitative data are expressed as mean ± standard error of the mean (SEM) from at least three independent experiments (biological replicates), with each measurement performed in triplicate (technical replicates) where applicable. Data were analyzed using GraphPad Prism 9 software (GraphPad Software, San Diego, CA, USA). Comparisons between the two groups were performed using an unpaired two‐tailed Student’s *t*‐test. For multiple group comparisons, one‐way ANOVA followed by Tukey’s post hoc test was applied. Differences were considered statistically significant at *p* < 0.05. For animal experiments, *n* = 8 mice per group. For histological and immunostaining analyses, three sections per mouse and five random fields per section were quantified, and the mean value for each mouse was used for statistical analysis. Cell‐based CCK‐8, SOD, and total antioxidant capacity assays were performed in three independent experiments with triplicate measurements, yielding *n* = 9 measurements per group. Western blot and Co‐IP were independently repeated three times.

## 3. Results

### 3.1. MitoQ Inhibits Cardiac Fibrosis and Improves Cardiac Function in Diabetic Mice

**Figure 1:**
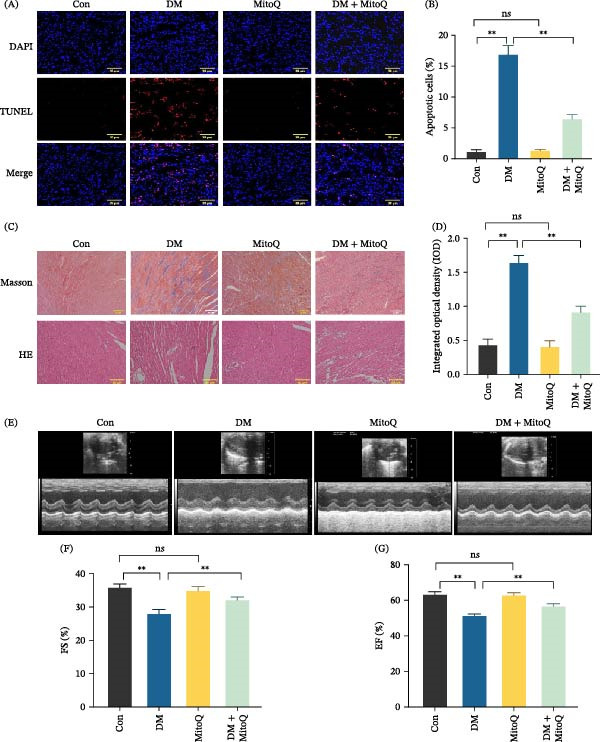
MitoQ improves the degree of cardiac fibrosis and inhibits the decline of cardiac function caused by diabetes. (A) TUNEL staining of mouse heart tissue (20×). (B) Quantitative analysis of TUNEL staining results of cardiac tissue. (C) HE staining and Masson staining of mouse heart tissue (20×). (D) Quantification of myocardial fibrosis based on Masson’s staining. (E) Representative echocardiographic images of mouse hearts. (F) Left ventricular fractional shortening (LVFS) in each group. (G) Left ventricular ejection fraction (LVEF) in each group. Data are presented as mean ± SEM. For animal experiments, *n* = 8 mice per group.  ^∗∗^ *p* < 0.05.

To determine whether diabetes induces myocardial injury, cardiomyocyte DNA fragmentation was first assessed by TUNEL staining. Compared with the control group, diabetic mice exhibited a marked increase in TUNEL‐positive nuclei, indicating enhanced cardiomyocyte death. Notably, MitoQ treatment significantly reduced the proportion of TUNEL‐positive cells (Figure 1A,B). Given that persistent cardiomyocyte loss often contributes to structural remodeling, histopathological changes were further examined. H&E staining revealed that long‐term hyperglycemia led to myocardial edema, disorganized cardiomyocyte arrangement, and structural disruption. Consistently, Masson’s trichrome staining demonstrated excessive interstitial collagen deposition in diabetic mice, confirming aggravated myocardial fibrosis. Importantly, MitoQ administration alleviated myocardial edema, improved cellular organization, and reduced collagen accumulation (Figure 1C,D), suggesting a protective effect against diabetes‐induced structural remodeling. Since structural remodeling is closely associated with cardiac dysfunction, echocardiographic evaluation was subsequently performed. Diabetic mice showed a significant decline in the left ventricular inner diameter shortening rate and ejection fraction, reflecting impaired systolic function. MitoQ treatment partially restored these functional parameters (Figure 1E–G). Together, these findings suggest that MitoQ mitigates diabetes‐induced cardiomyocyte death and adverse remodeling, thereby improving cardiac function in diabetic mice.

### 3.2. MitoQ Inhibits the TXNIP/NLRP3 Pathway and Improves Oxidative Stress in the Hearts of Diabetic Mice

**Figure 2:**
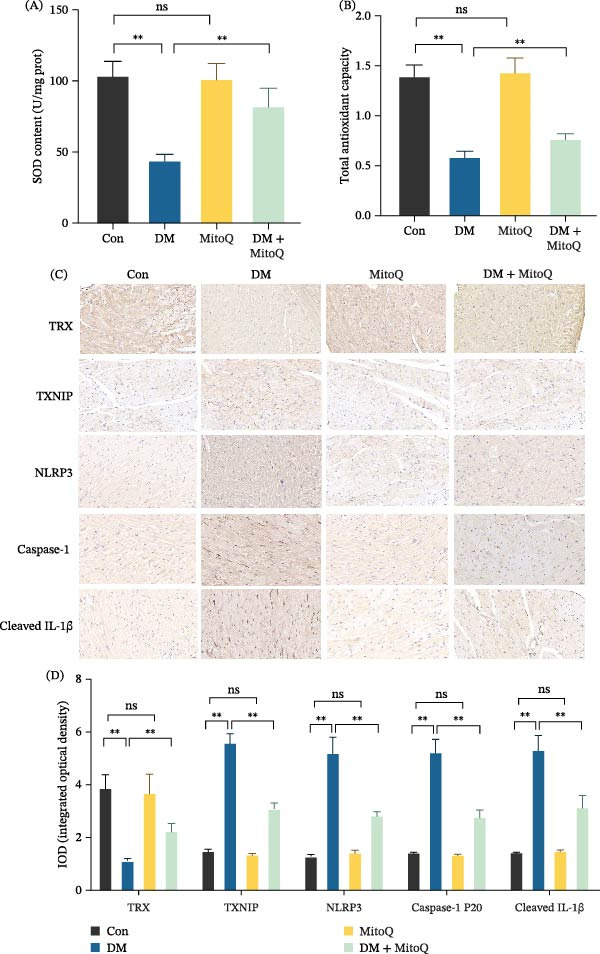
MitoQ alleviates oxidative stress and inhibits activation of the TXNIP/NLRP3 pathway in diabetic mouse hearts. (A) Detection of total SOD content in mouse myocardial tissue. (B) Detection of total antioxidant capacity in mouse myocardial tissue. (C) Immunohistochemical detection of TRX, TXNIP, NLRP3, Caspase‐1, and cleaved IL‐1β protein expression in mouse heart tissue (20×). (D) Quantitative analysis of immunohistochemical staining results. Data are presented as mean ± SEM. For animal experiments, *n* = 8 mice per group.  ^∗∗^ *p* < 0.05.

To assess oxidative stress in the myocardial tissue, total SOD content and total antioxidant capacity were measured. Compared with the control group, diabetic mice exhibited significantly reduced SOD levels and total antioxidant capacity, indicating increased oxidative stress in the heart. MitoQ treatment restored these antioxidant parameters toward normal levels (Figure 2A,B). Immunohistochemical analysis was performed to examine the expression of proteins related to the TXNIP/NLRP3 signaling pathway. In diabetic hearts, the expression of TXNIP, NLRP3, Caspase‐1, and cleaved IL‐1β was markedly increased, whereas TRX expression was decreased. Treatment with MitoQ substantially attenuated these changes, suggesting that MitoQ can suppress the activation of the TXNIP/NLRP3 pathway and mitigate pyroptotic signaling in the diabetic myocardium (Figure 2C,D).

### 3.3. MitoQ Inhibits ROS Production in Mitochondria of AC16 Cardiomyocytes Under High‐Glucose Conditions

**Figure 3:**
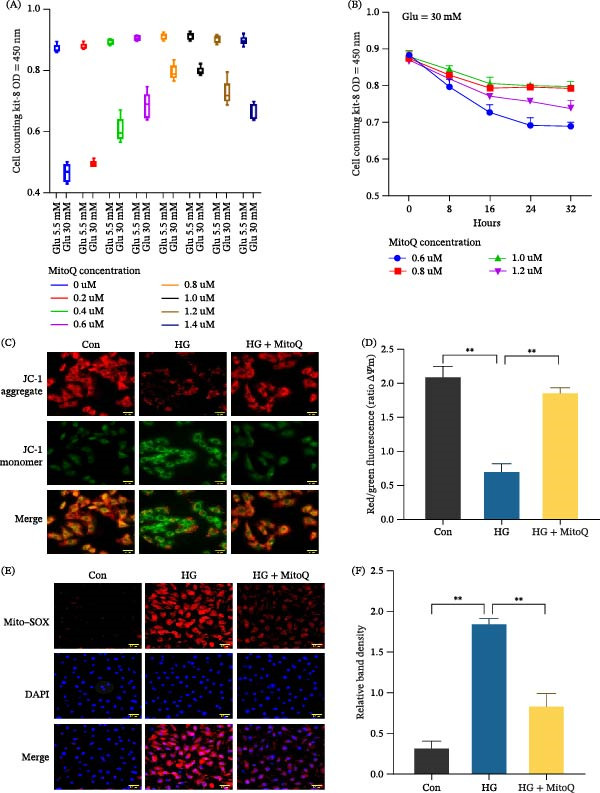
MitoQ reduces mitochondrial ROS production in AC16 cardiomyocytes under high‐glucose conditions. (A) CCK‐8 assay showing cell viability of AC16 cardiomyocytes treated with different concentrations of MitoQ under 5.5 mM (normal glucose) and 30 mM (high glucose) conditions. (B) Time‐dependent effects of different concentrations of MitoQ on AC16 cell viability under high‐glucose conditions, assessed by CCK‐8 assay. (C) Representative images of mitochondrial membrane potential in AC16 cells (20×). (D) Quantification of mitochondrial membrane potential fluorescence intensity. (E) Representative images of mitochondrial ROS production in AC16 cells (20×). (F) Quantitative analysis of mitochondrial ROS fluorescence intensity. Data are presented as mean ± SEM. CCK‐8 assays were performed with *n* = 9 measurements per group.  ^∗∗^ *p* < 0.05.

**Figure 4:**
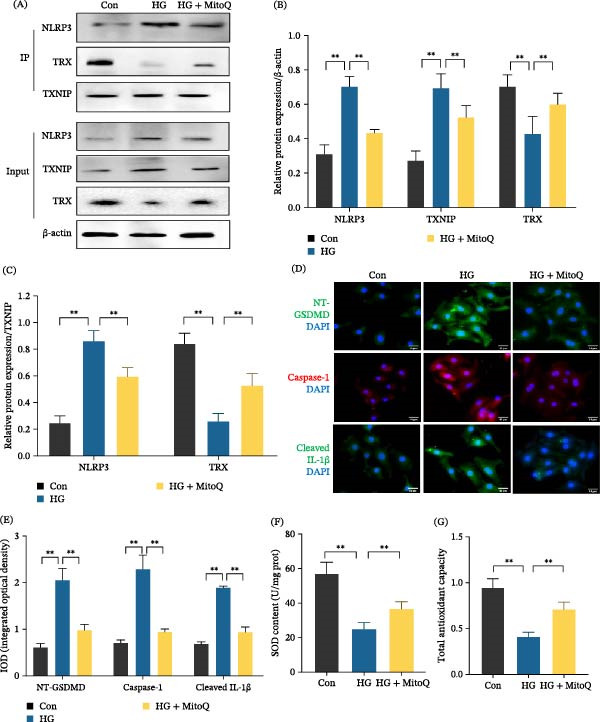
MitoQ inhibits the activation of NLRP3 inflammasome in AC16 cells under high‐glucose conditions. (A) Representative co‐immunoprecipitation (Co‐IP) and western blot analyses showing the interaction among TRX, TXNIP, and NLRP3 proteins in AC16 cells. (B) Quantitative analysis of TRX, TXNIP, and NLRP3 protein expression levels in the input group. (C) Quantitative analysis of TRX–TXNIP and TXNIP–NLRP3 interactions in the IP group. (D) Representative immunofluorescence images of NT ‐GSDMD, Caspase‐1, and cleaved IL‐1β expression in AC16 cells. (E) Quantitative analysis of immunofluorescence intensity. (F) Total SOD content in AC16 cells. (G) Total antioxidant capacity in AC16 cells. Data are presented as mean ± SEM; WB and Co‐IP, *n* = 3 independent experiments; SOD and total antioxidant capacity assays, *n* = 9 measurements per group.  ^∗∗^ *p* < 0.05.

To determine the optimal intervention conditions, AC16 cell viability was assessed using the CCK‐8 assay. The results showed that cell viability was highest when cells were pretreated with 1 μM MitoQ for 24 h under high‐glucose conditions (Figure 3A,B). 1 μM MitoQ was selected for subsequent experiments. Given that mitochondrial dysfunction is a major source of oxidative stress under hyperglycemic conditions, MMP and intramitochondrial ROS levels were examined. High‐glucose stimulation induced significant MMP depolarization and mtROS accumulation in AC16 cardiomyocytes. MitoQ treatment markedly attenuated MMP damage (Figure 3C,D) and reduced mtROS production (Figure 3E,F). To further investigate the underlying mechanism, the expression of key proteins involved in the TXNIP/NLRP3 pathway was analyzed by western blot. High‐glucose exposure decreased TRX expression while increasing TXNIP and NLRP3 levels. MitoQ treatment reversed these alterations (Figure 4A,B). Co‐immunoprecipitation assays were subsequently performed to evaluate protein–protein interactions. High‐glucose stimulation promoted the dissociation of TRX from TXNIP and enhanced the interaction between TXNIP and NLRP3 (Figure 4A,C), indicating the activation of the inflammasome pathway. Consistently, activation of NLRP3 led to increased expression of downstream pyroptosis‐related proteins, including NT‐GSDMD, Caspase‐1, and cleaved IL‐1β (Figure 4D,E). MitoQ intervention inhibited the TXNIP–NLRP3 interaction and suppressed the upregulation of these downstream proteins. In addition, MitoQ treatment restored the total SOD content and total antioxidant capacity in cardiomyocytes under high‐glucose conditions (Figure 4F,G), further confirming its antioxidative effect. Taken together, these findings demonstrate that MitoQ suppresses high‐glucose‐induced NLRP3 activation by inhibiting the mtROS/TXNIP signaling pathway, thereby reducing pyroptotic signaling in cardiomyocytes.

### 3.4. TXNIP Knockdown Attenuates High‐Glucose‐Induced Cardiomyocyte Injury

**Figure 5:**
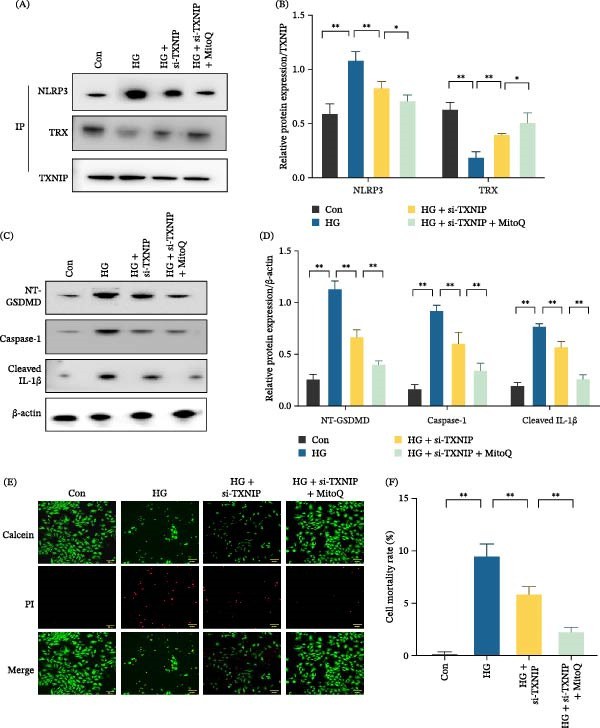
Combined TXNIP knockdown and MitoQ treatment attenuates high glucose‐induced NLRP3 inflammasome activation and cardiomyocyte death. (A) Representative co‐immunoprecipitation (Co‐IP) analysis showing the interaction among TRX, TXNIP, and NLRP3 proteins in AC16 cells. (B) Quantitative analysis of TRX –TXNIP and TXNIP–NLRP3 interactions in the IP group. (C) Representative western blot analysis of NT‐GSDMD, Caspase‐1, and cleaved IL‐1β expression in AC16 cells. (D) Quantitative analysis of western blot results. (E) Representative propidium iodide (PI) staining images showing cardiomyocyte death. (F) Quantitative analysis of PI test results. Data are presented as mean ± SEM; WB and Co‐IP, *n* = 3 independent experiments.  ^∗∗^ *p* < 0.05.

To determine whether MitoQ improves myocardial damage caused by high glucose through the TXNIP/NLRP3 pathway, we interfered with the expression of the TXNIP protein in AC16 cells. Experimental results show that interfering with TXNIP expression reduces the interaction between TXNIP and NLRP3 proteins (Figure 5A,B) and reduces the expression of NT‐GSDMD, Caspase‐1, and cleaved IL‐1β proteins in a high‐glucose environment (Figure 5C,D). The results of the PI experiments showed that interfering with TXNIP expression reduced the death rate of cardiomyocytes in a high‐glucose environment (Figure 5E,F). In addition, under high‐glucose conditions, combined reduction of TXNIP expression and MitoQ treatment further decreased the expression levels of NT‐GSDMD, Caspase‐1, and cleaved IL‐1β and increased the SOD content and total antioxidant capacity compared with either intervention alone (Figure S1A–S1C). These findings suggest that combined reduction of TXNIP expression and MitoQ treatment further attenuates high glucose‐induced NLRP3 inflammasome activation, oxidative stress, and cardiomyocyte death without implying a synergistic interaction.

## 4. Discussion

Oxidative stress is currently considered the primary pathogenesis of DCM; therefore, antioxidant treatment of DCM is critical. Previous studies have shown that ROS, DNA damage, potassium efflux, and lysosomal damage can activate the NLRP3 inflammasome and induce cell pyroptosis [16, 20]. During oxidative stress, impaired electron transfer within mitochondrial respiratory chain complexes I and III leads to excessive production of mtROS [21, 22]. The accumulation of mtROS disrupts mitochondrial function and contributes to cardiomyocyte injury. In addition, mtROS has been reported to promote NLRP3 deubiquitination, thereby facilitating inflammasome activation [23]. In the present study, both in vivo and in vitro experiments demonstrated that high‐glucose stimulation activated the NLRP3 inflammasome in cardiomyocytes and significantly increased the expression of downstream proteins, including NT‐GSDMD, Caspase‐1, and cleaved IL‐1β. Treatment with the mitochondria‐targeted antioxidant MitoQ reduced mtROS production, decreased TXNIP–NLRP3 interaction, and attenuated NLRP3 inflammasome activation. These findings suggest that mtROS plays a critical role in mediating TXNIP/NLRP3 pathway activation in DCM. Therefore, preserving mitochondrial redox homeostasis and inhibiting TXNIP/NLRP3 signaling may represent viable therapeutic strategies for DCM.

Activation of the NLRP3 inflammasome is a central event in pyroptosis and contributes to the progression of diabetic complications, including DCM. Previous studies have shown that inhibition of NLRP3 activation alleviates diabetes‐induced myocardial injury [24, 25]. However, the upstream mechanisms responsible for inflammasome activation were not fully elucidated. TXNIP, an endogenous inhibitor of the TRX system, has been identified as a key mediator linking oxidative stress to NLRP3 activation in diabetes‐related complications and cardiovascular diseases. In diabetic retinopathy, vascular injury, and diabetes‐associated cerebrovascular disorders, reducing ROS levels suppresses TXNIP–NLRP3 binding and ameliorates tissue damage [26, 27]. In heart diseases, inhibiting the interaction between TXNIP and NLRP3 can reduce the progression of myocardial ischemic damage and doxorubicin‐induced myocardial damage [28, 29]. Our study further demonstrated that the mtROS/TXNIP/NLRP3 signaling axis is activated in DCM and contributes to cardiomyocyte injury and cardiac dysfunction. Both MitoQ treatment and TXNIP knockdown weakened the activation of this pathway and reduced cardiomyocyte injury under hyperglycemic conditions. These findings support a mechanistic link between mtROS accumulation and TXNIP‐mediated NLRP3 activation in DCM.

Excessive mtROS accumulation disrupts the MMP, aggravates mitochondrial dysfunction, and promotes inflammatory responses [30]. MitoQ, a mitochondria‐targeted antioxidant, selectively accumulates within mitochondria and scavenges excess mtROS, thereby exerting anti‐inflammatory and cytoprotective effects [31]. Previous studies have shown that MitoQ alleviates lung injury and myocardial ischemia–reperfusion injury by reducing intracellular ROS production [32, 33]. Consistent with these findings, our results demonstrated that MitoQ inhibited the TXNIP–NLRP3 interaction and attenuated cardiomyocyte injury under high‐glucose conditions. Furthermore, TXNIP knockdown enhanced the protective effect of MitoQ, highlighting the importance of the mtROS/TXNIP/NLRP3 pathway in diabetic myocardial injury.

## 5. Conclusion

In summary, activation of the mtROS/TXNIP/NLRP3 signaling pathway plays a pivotal role in diabetic myocardial injury. mtROS accumulation promotes TXNIP binding to NLRP3, leading to inflammasome activation and subsequent cardiomyocyte injury and cardiac dysfunction. MitoQ inhibits mtROS generation, suppresses NLRP3 activation, mitigates hyperglycemia‐induced myocardial damage, and preserves cardiac function. These findings provide new insights into the pathogenesis of DCM and suggest potential therapeutic targets for its treatment.

## Author Contributions

Conceptualization: Wei Zhang, Junjie Leng, and Chao Shi. Methodology: Wei Zhang, Junjie Leng, and Zhuoya Yao. Validation: Junjie Leng and Chao Shi. Investigation: Junjie Leng and Shaohuan Qian. Writing – original draft preparation: Wei Zhang. Writing – review and editing: Chao Shi and Shaohuan Qian. Supervision: Chao Shi, Shaohuan Qian, and Zhuoya Yao.

## Funding

This study was supported by the Natural Science Foundation of Bengbu Medical University (Grant 2023byzd074).

## Disclosure

All authors have read and agreed to the published version of the manuscript.

## Conflicts of Interest

The authors declare no conflicts of interest.

## Supporting Information

Additional supporting information can be found online in the Supporting Information section.

## Supporting information


**Supporting Information** The Supporting information Appendix accompanying this article contains the following material: Figure S1, TXNIP knockdown combined with MitoQ treatment attenuates high glucose‐induced cardiomyocyte pyroptosis and enhances antioxidant capacity.

## Data Availability

The raw data supporting the conclusions of this article will be made available by the authors without undue reservation.
