# Supplementary material for: MitoQ Ameliorates Diabetic Cardiomyopathy by Inhibiting the mtROS–TXNIP–NLRP3 Pathway
Source: Mediators Inflamm. 2026 Jul 21;2026:1505350. doi: 10.1155/mi/1505350 (PMC13387309; doi:10.1155/mi/1505350)
Supplement: Supplementary file 1 — Supporting Information The Supporting information Appendix accompanying this article contains the following material: Figure S1, TXNIP knockdown combined with MitoQ treatment attenuates high glucose‐induced cardiomyocyte pyroptosis and enhances antioxidant capacity. [file MI-2026-1505350-s001.docx]

**Supplementary Material**

Under high-glucose conditions, compared with MitoQ treatment alone or TXNIP knockdown alone, combined TXNIP knockdown and MitoQ treatment further decreased the protein expression levels of NT-GSDMD, Caspase-1, and cleaved IL-1β (Fig S1A). Meanwhile, analysis of oxidative stress-related indicators showed that, compared with MitoQ treatment alone or TXNIP knockdown alone, combined TXNIP knockdown and MitoQ treatment further increased intracellular SOD content and total antioxidant capacity in cardiomyocytes (Fig S1B，S1C).

| 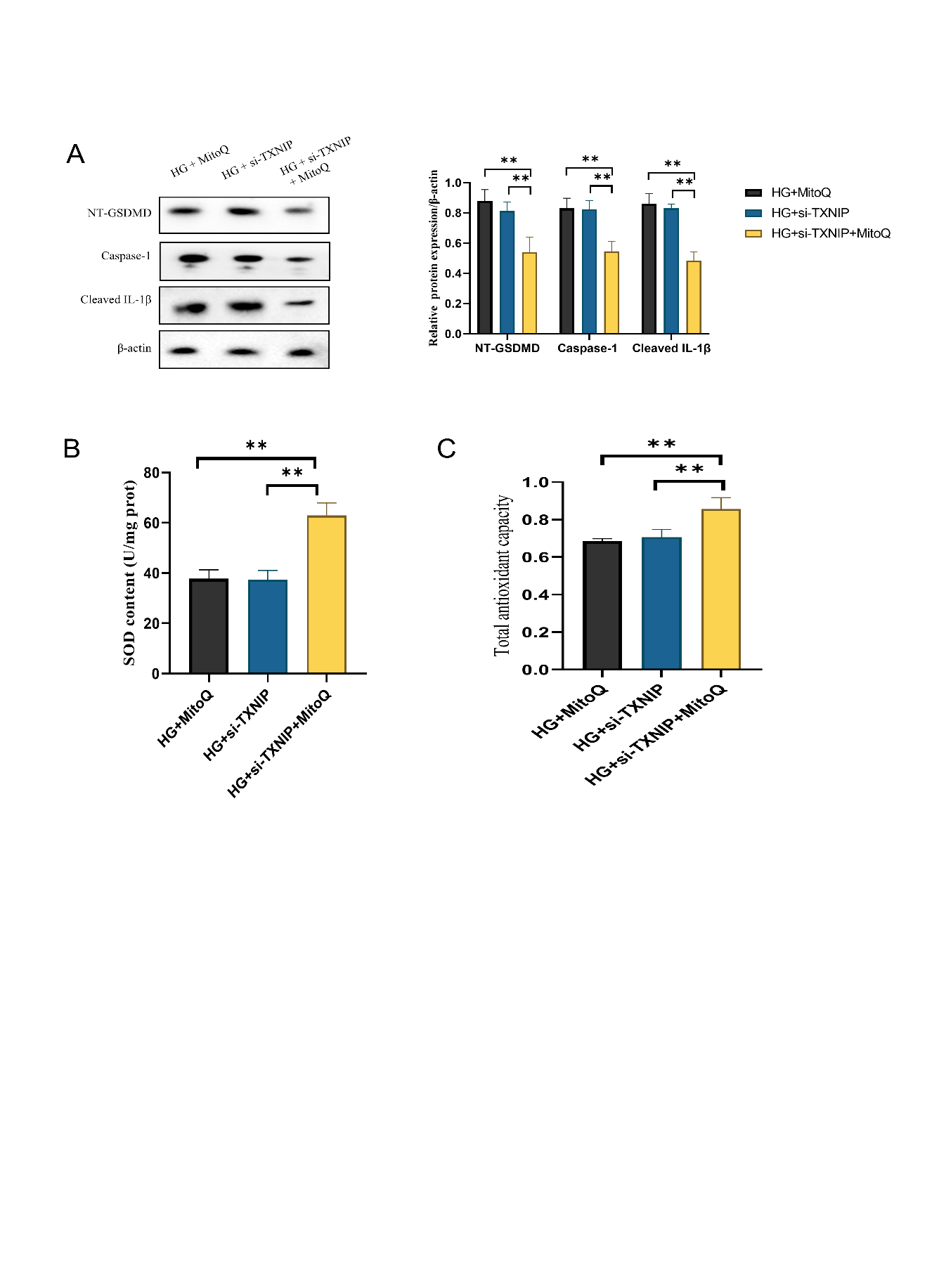 |
| --- |

**Fig S1. TXNIP knockdown combined with MitoQ treatment attenuates high glucose-induced cardiomyocyte pyroptosis and enhances antioxidant capacity.**

**A.**Western blot analysis of NT-GSDMD, Caspase-1, and cleaved IL-1β protein expression in cardiomyocytes from each group.**B.**Measurement of SOD content in cardiomyocytes from each group.**C.** Measurement of total antioxidant capacity in cardiomyocytes from each group. Data are presented as mean ± SEM. Western blot analysis was independently repeated three times. SOD and total antioxidant capacity assays were performed with n = 9 measurements per group.
